# Genetic Control of Spontaneous Arthritis in a Four-Way Advanced Intercross Line

**DOI:** 10.1371/journal.pone.0075611

**Published:** 2013-10-11

**Authors:** Laura Mellado Ranea, Andreia de Castro Marques, Steffen Möller, Yask Gupta, Saleh M. Ibrahim

**Affiliations:** Department of Dermatology, University of Lübeck, Lübeck, Germany; University Hospital Jena, Germany

## Abstract

Identifying the genetic basis of complex diseases, such as rheumatoid arthritis, remains a challenge that requires experimental models to reduce the genetic and environmental variability. Numerous loci for arthritis have been identified in induced animal models; however, few spontaneous models have been genetically studied. Therefore, we generated a four-way advanced intercross line (AIL) from four inbred strains, including BXD2/TyJ which spontaneously develops autoimmune arthritis. A genome-wide scan for spontaneous arthritis was performed in a cohort of 366 mice of the fourth generation (G4) of this cross. Five loci contributing to clinical phenotypes were identified in chromosomes 3, 7, 13, 18, and X. Three of the loci found in this study, confirm previously identified loci; whereas two of them are novel loci. Interesting candidate genes for the loci are highlighted. This study provides a genetic overview of spontaneous arthritis in mice and aids to solve the genetic etiology of rheumatoid arthritis and to gain a better understanding of the disease.

## Introduction

Rheumatoid arthritis (RA) is a systemic chronic autoimmune inflammatory disease that primarily affects joints. Genetic factors have a strong impact in RA susceptibility and development [1]. The heritability of RA is estimated to be around 60% [2]. The most important genetic risk is the human leukocyte antigen (HLA) locus. Its influence is estimated to be one third of the overall genetic susceptibility to RA [2], but it confers susceptibility only to autoantibodies to citrullinated protein antigens (ACPA)-positive patients [3].

The knowledge of genes and pathways involved in disease is of great importance to understand the pathogenic mechanisms of disease, and consequently to improve therapy, diagnosis and disease prevention. Linkage and association studies in human are commonly used to identify candidate susceptibility loci in Mendelian disorder. However, the heterogeneity of the human genome, the minor single gene contribution to the pathogenesis, and the multiplex gene-gene and gene-environment interactions render challenging to identify genes with low effect in complex disease. Therefore, animal models are invaluable tools to decipher genetic factors affecting quantitative traits, since they allow us to control the genetic background and to define the environmental conditions.

BXD2/TyJ is a recombinant inbred strain generated by inbreeding for more than 20 generations a F2 intercross of the strains C57BL/6J and DBA/2J [4]. BXD2/TyJ mice spontaneously develop chronic erosive arthritis and generalized autoimmune disease. Mice start to develop arthritis after 4 months, and between 9 and 12 months 66% of females and 42% of males are affected. BXD2/TyJ mice produce high titers of rheumatoid factor (RF) and antibodies against DNA, with predominance of IgG1 and IgG2b isotypes. Those mice also demonstrate glomerulonephritis, proteinuria, and splenomegaly [5]. BXD2/TyJ strain develops features of autoimmune diseases due to a complex combination of interacting genes inherited from the original parental strains, C57BL/6J and DBA/2J, which develop neither arthritis nor lupus. These characteristics make the BXD2/TyJ strain an exceptional model to study genetics of autoimmune diseases such as erosive arthritis.

There are different strategies to identify loci controlling quantitative traits (so called quantitative trait loci, QTLs) in murine animal model. The traditional mapping in a cross between two inbred strains (F2 intercross and N2 backcross) is restricted by the low resolution and the limited genetic variability. An advanced intercross line (AIL) is produced by random and sequential intercrossing of two or more inbred strains for many generations by avoiding brother–sister mating, so that the progeny carries a recombinant genome from the parental strains [6]. An AIL offers a higher genomic resolution than conventional F2 and N2 crosses due to the accumulation of a greater density of recombination events, and therefore, is a powerful approach to identify and further refine QTLs.

Here, the genetic determinants of the BXD2/TyJ strain were studied in a four-way autoimmune-prone AIL generated from four inbred strains. A whole-genome scan was performed in a cohort of mice from the four generation of the AIL, and several genomic regions controlling different phenotypes were identified.

## Materials and Methods

### Mice

An outbred four-way autoimmune-prone advanced intercross line (AIL) was generated by our group from the parental mouse strains BXD2/TyJ, MRL/MpJ, NZM2410/J and CAST/EiJ, all acquired from The Jackson Laboratory (Bar Harbor, ME). The four inbred strains were intercrossed following an equal strain and sex distribution. At each time, 50 breeding pairs were used to generate the next generation. A total of 366 mice from the fourth generation (G4) were enrolled in this study and evaluated for 6 months. Mice were assessed once a week to evaluate clinical disease according to a scoring system based on the number of inflamed joints [7]. Each paw was scored individually, each inflamed toe and knuckle was given a score of 1, and an inflamed wrist or ankle was given a score of 5; maximum score of 15 per limb and of 60 per mouse. Mice were bred and housed under climate-controlled conditions with 12-h light/darkness cycles at the animal facility at the University of Rostock. The procedures were approved by the governmental administration of the State of Mecklenburg-Vorpommern.

### Genotyping

Pure genomic DNA was isolated with the DNeasy Blood & Tissue Kit (Qiagen, Hilden, Germany) according to manufacturer's instructions. Illumina Mouse MD Linkage Panel was used to genotype 1,449 SNPs, of which 1,199 were polymorphic between the parental strains. The average distance between informative markers was 1.77 Mbp.

### QTL mapping

#### Single locus linear model

The association analysis was performed by the R version of HAPPY [8] on Debian Linux [9]. In brief, the founder haplotype structure for each mouse is inferred by the HAPPY algorithm taking into account the adjacent markers. The association seeks for differences between the genetic effects of the parental haplotypes. QTLs then are detected by a regression model applied to the inferred haplotypes. This association provides ANOVA significance levels, presented as the negative base-10 logarithmic *P* value (-log *P*). To determine the empirical threshold for statistical significance, 1,000 permutations were performed [8]. An empirical significance threshold was established at *P*<0.001 for each phenotype. Data from all chromosomes were analyzed simultaneously with an additive model.

Some of the mice in this study were simultaneously assessed for autoimmune skin blistering disease (epidermolysis bullosa acquisita, EBA) development for an independent study, and therefore immunized with type VII collagen (ColVII) [10]. To exclude a bias in the analysis, ColVII immunization and sex were included as covariates. Confidential intervals (CI) of QTLs were estimated manually to comprise the region around a peak up to a drop of the *P* value by *P*<0.05.

#### Efficient mixed-model

To correct for family structure and genetic relatedness among mice and reduce the inflation of false positives, a variance component model approach was used. The Efficient Mixed-Model Association eXpedited (EMMAX) beta version [11] was used for testing association between individual markers and the phenotype. This software works in a computationally efficient manner by avoiding repetitive variance component estimation methods, since it considers that each locus explains only a small fraction of the complex trait. Therefore, a kinship matrix is generated by using Balding-Nicholas procedure in emma-kin function, and then the matrix is used as additional random variable while performing the SNP associations.

Genotype and phenotype of each mouse are provided in Table S1.

## Results

### The G4 mice of the four-way autoimmune-prone advanced intercross line developed spontaneous arthritis

The outbred four-way autoimmune-prone advanced intercross line (AIL) was originated in our group from the parental mouse strains BXD2/TyJ, MRL/MpJ, NZM2410/J and CAST/EiJ. In the fourth generation (G4), 366 mice were monitored to evaluate clinical arthritis for 6 months. G4 mice developed spontaneous arthritis in 85.8% of males and 57.3% of females. Onset of disease, which reflects the speed of disease progression, and severity of disease, which is measured as maximum score, were similar in both genders (Table 1). A lower percentage of these mice also developed autoimmune pancreatitis [12] and lupus nephritis (data not published).

**Table 1:** Phenotypic characteristics of spontaneous arthritis in G4 mice from the four-way autoimmune-prone AIL.

	Incidence	Onset^a^	Maximum score^b^
Females	113/197	22.9 ± 0.4	4.5 ± 0.4
Males	145/169	21.9 ± 0.4	6.9 ± 0.4
Total	258/366	22.3 ± 0.3	5.6 ± 0.3

a. Onset measured in weeks. Mean ± standard error of the mean; only disease mice were included in the calculation.

b. Mean ± standard error of the mean; all mice were included in the calculation.

### Five loci associated with arthritis phenotypes in the G4 of the four-way autoimmune-prone advanced intercross line were identified

**Figure 1:**
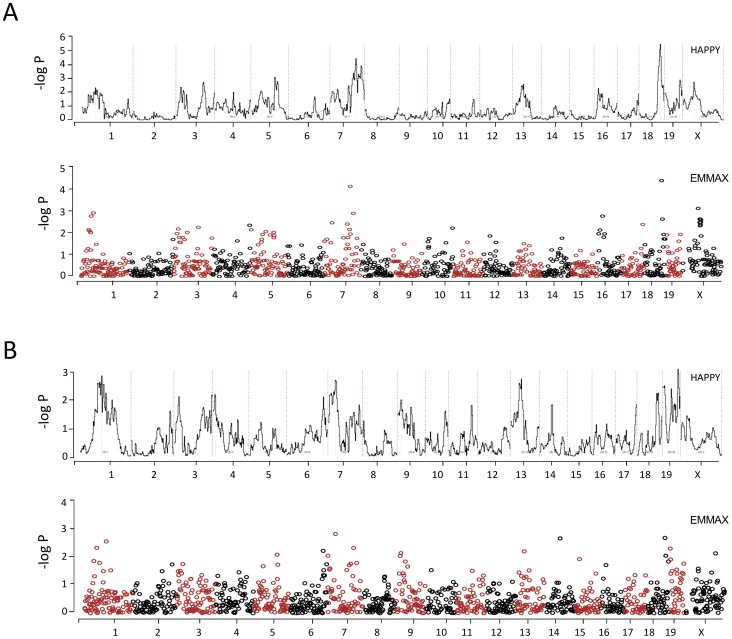
Whole-genome association map for spontaneous arthritis traits. Phenotypes are (A) susceptibility to disease, and (B) disease severity, measured as maximum score. Each graph represents the strength of association between phenotype and marker, using HAPPY (linear plot) or EMMAX (Manhattan plot), for the whole set of 366 G4 mice tested. Gender and ColVII immunization were used as covariates. The x-axis indicates the SNP's chromosomal position, and the y-axis shows the -log *P* value of association.

The genetic control of susceptibility and maximum score was analyzed by an association study performed with the software package HAPPY. Results are represented in Figure 1. Statistical significance was based on ANOVA test (-log *P*), and correspond nicely with the empirical significance levels as assessed by 1,000 permutations for every SNP. A cutoff of 0.001 in the empirical *P* value, equivalent to one false positive per 1,000 associated SNPs, was defined and those SNPs above this threshold were considered significant. Empirical *P* values <0.01 were considered suggestive.

HAPPY is the method commonly used for association studies in heterogeneous stocks. It associates haplotypes from the parental strains with the phenotypes and is considered more powerful than single-point methods to identify loci in complex murine crosses since provides information from the parental strains [8]. However, this method does not take into account the genetic relatedness of the mice. It is known that in complex mice crosses as AILs and heterogeneous stocks, family structure and genetic relatedness are important source of false positive findings [13]. Mixed models have the power to effectively exclude relatedness from the analysis. Therefore, we used EMMAX [11], a modification of EMMA which have been successfully used to identify loci in previous human and animal studies, to confirm the loci identified by HAPPY and to exclude false positives. This method associates individual SNPs, instead of haplotypes as HAPPY, with the phenotype, and has the property of include kinship as variable. Statistical significance was also assessed by permutation assay. Loci which reached significance level by both methods were considered significant. Loci with high association by one of the methods and suggestive association in the other were considered suggestive.

Five loci associated with susceptibility or maximum score were found in the screen (Table 2). Two loci were significantly associated with susceptibility to disease on chromosomes 7 and 18, and two loci showed suggestive evidence of association with the same phenotype on chromosome 3 and X (Figure 1 A). Maximum score showed suggestive evidence of association to one locus on chromosomes 13 (Figure 1 B). The loci on chromosome 7, 18 and X were located in previously mapped arthritis QTLs (Table 2); while the locus on chromosome 3 spanned a region without previously mapped autoimmune QTLs. The chromosome 13 QTL did not overlap with known arthritic loci, but with loci controlling disease in models of other human autoimmune diseases.

**Table 2:** Identified loci for susceptibility and maximum score.

Chr	Phenotype	HAPPY peak	Position (Mbp^a^)	Peak -log *P*	Confidential interval	EMMAX peak	Peak -log *P*	Published arthritic loci	Ref.
18	Susceptibility	rs13483436	64,67	5.4*	rs3688789–rs13483466	rs6161154	4.4*	*Pgia11, Cia18*	[17]
7	Susceptibility	rs3707067	105,86	4.4*	rs6213614–rs3716088	rs3713052	4.1*	*Cia7, Pgia3, Pgia21*	[24] [18] [17]
X	Susceptibility	rs3157124	68,69	2.7#	rs13483765–rs3725966	rs13483825	3.1*	*Pgia24*	[17]
3	Susceptibility	rs3659988	16,35	2.3*	rs6248752–rs6235984	rs6235984	2.2#		
13	Max. score	rs13481764	36,64	2.6*	rs6275055–rs13481783	rs3725187	2.2#		

a. Position according to the NCBI Build 37.

* Empirical *P*<0.001; # empirical *P*<0.01.

Chr, chromosome; Mbp, megabase pair; max. score, maximum score.

## Discussion

RA is considered rather a syndrome than a discrete disease with a single etiologic source [14]. In fact, RA has already been divided according to the presence or absence of ACPA [15]. This division is also reflected in the genetic heterogeneity and clinical phenotype: HLA-DRB1 and PTPN22 loci are exclusively associated with ACPA-positive patients who, in addition show more severe and destructive disease than ACPA-negative patients [16]. It is noteworthy that BXD2/TyJ mice present RF in peripheral blood. Together with the fact that arthritis develops spontaneously, this strain may be considered a specific model for autoantibody-positive RA.

In this study, the most extensive genetic analysis of BXD2/TyJ spontaneous arthritis was generated. Five arthritis loci were identified, of which two had not been implicated in any previous genetic studies in arthritis.

The family structure of complex crosses such AILs complicates the association analysis, potentially leading to many false positive findings. Therefore, we used a software to account for relatedness. Although no QTL was fine-mapped in sufficient detail to identify the causal genetic variant, potential candidate genes within the loci are highlighted below.

One of the most strongly associated loci maps towards the telomeric end of chromosome 18 and controls susceptibility to disease. This locus spans the previously identified *Pgia11* and *Cia18* loci, which are associated with susceptibility to arthritis and autoantibody production, respectively [17], [18]. This locus also overlaps with loci associated with the murine models of multiple sclerosis (*Eae25*) [19], systemic lupus erythematosus (*Lbw6*) [20], type I diabetes (*Idd21.1*) and autoimmune ankylosing spondylitis (*Pgis1*) [21]. The importance of chromosome 18 in susceptibility to autoimmunity in different species had already been reported [22]. In fact, the QTL identified in this study contains genes whose human orthologous had previously been associated with RA in a genome-wide association study (GWAS) [23] such as *PTPN2* (protein tyrosine phosphatase, non-receptor type 2, lymphoid), *TCF4* (transcription factor 4), *ZBTB7C* (zinc finger and BTB domain containing 7C), *IMPA2* (inositol(myo)-1(or 4)-monophosphatase 2), *ATP9B* (ATPase class II type 9B or macrophage MHC receptor 1), *DYM* (dymeclin), *CTIF* (CBP80/20-dependent translation initiation factor), and *CCDC11* (coiled-coil domain containing 11). More compelling candidates within the locus are *Nfatc1* gene encoding calcineurin-dependent nuclear factor 1 of activated T cells; *Smad2*, *Smad7* and *Smad4* genes encoding proteins from the SMAD (similar to mothers against decapentaplegic) family which mediates TGF-β signaling; *Dcc* gene encoding a netrin 1 receptor, a member of the immunoglobulin superfamily of cell adhesion molecules; and *Malt1* gene encoding a caspase-like protein involved in B-cell and T-cell receptor signaling pathways.

On chromosome 7, a locus strongly associated with susceptibility was found. This locus overlaps with *Cia7* [24], *Pgia3* [18], and *Pgia21* [17], known loci which contribute to induced arthritis; and with loci controlling other experimental autoimmune disease such as *Eae4* [25], *Nba3* [26], *Eae26* [27], and *Il4ppq* [28]. This QTL has also conservation of synteny with a human region containing genes associated with arthritis such as *PRKCB1* (protein kinase C, beta 1) and *PDE2A* (phosphodiesterase 2A, cGMP-stimulated) [29]. Other candidate genes of particular interest within the locus are the genes encoding the interleukins IL18BP, IL21 and IL27.

On chromosome X, one locus had suggestive association with susceptibility to disease. This association explains at least part of the strong sex effect on arthritis susceptibility. Accordingly, the phenotype maximum score for which no sex effect was found, did not show association with this locus. The region matches with the previously mapped genetic locus *Pgia24* controlling antibody response [17]. Plausible biological candidates are *Ikbkg* gene encoding the NF-κB essential modulator, NEMO, which regulates the activation of NF-κB; and *Irak1* gene encoding the interleukin-1 receptor-associated kinase 1.

A locus on chromosome 3 was suggestively associated with susceptibility to disease. This novel QTL was not previously associated with any autoimmune phenotype; however, it harbors potential candidate genes such as *Pde7a* which encodes a protein from the cyclic nucleotide phosphodiesterase (PDE) family with a relevant role in immune cell activation [30].

The locus that showed suggestive association with maximum score on chromosome 13 overlaps with two loci, *Bxs6* [31] and *Idd14* [32], controlling different clinical phenotypes in model of autoimmune nephritis and type I diabetes, respectively. The human corresponding region contained the gene *CDKAL1* which was associated with RA in a GWAS (rs1459047, [33]). Interesting genes within the locus are *Bmp6* coding a protein from the bone morphogenetic protein (BMP) family with known function in cartilage and bone formation and possible role in B-cell differentiation to plasmablasts [34]; and RIPK1 coding a serine-threonine kinase involved in necroptosis and inflammation [35].

The unique integration of genomes from four strains may explain the observation of two novel QTLs for arthritis, i.e. not found previously in other crosses, among the five identified (Table 2). On the other hand, our results show that it is likely that there are common pathways involved in different autoimmune diseases, since some of the QTLs identified in this study overlap with loci controlling autoimmune phenotypes in other mouse models.

The fact that human loci associated with RA in GWAS had corresponding loci in this cross indicates that it is likely that there are important common genes and pathways involved in arthritis in both humans and animals, particularly in the BXD2/TyJ strain. This is robust evidence to confirm that the approach followed in this study is appropriate and powerful to study genetic factor determining human RA in a hypothesis-free manner.

In summary, the present study demonstrates the utility of the generated four-way autoimmune-prone AIL to identify loci affecting arthritis. Two QTLs significantly associated with clinical arthritis and three QTLs with a suggestive association were successfully identified. This study confirms QTLs previously found in other arthritis models and identifies new risk loci for experimental arthritis. Further, fine mapping within each QTL combined with functional studies will be required to identify the causal genes and the pathways leading to disease.

## Supporting Information

Table S1 **Genotype-phenotype of the G4 mice of the four-way autoimmune-prone advanced intercross.**
(XLSX)
